# Laser fragmentation of amorphous and crystalline selenium of various morphologies and assessment of their antioxidant and protection properties

**DOI:** 10.3389/fchem.2024.1459477

**Published:** 2024-08-09

**Authors:** Alexander V. Simakin, Ilya V. Baimler, Anastasia O. Dikovskaya, Dina V. Kazantseva, Denis V. Yanykin, Valery V. Voronov, Oleg V. Uvarov, Maxim E. Astashev, Ruslan M. Sarimov, Vladimir E. Ivanov, Vadim I. Bruskov, Valeriy A. Kozlov

**Affiliations:** ^1^ Prokhorov General Physics Institute of the Russian Academy of Sciences, Moscow, Russia; ^2^ Institute of Theoretical and Experimental Biophysics of the Russian Academy of Sciences, Pushchino, Russia

**Keywords:** laser ablation, laser fragmentation, laser-induced breakdown, selenium nanoparticles, amorphous selenium, crystalline selenium, selenium nanorods

## Abstract

**Introduction:** The process of laser-induced breakdown of amorphous and crystalline selenium nanoparticles (Se NPs) of various shapes during nanosecond laser fragmentation of aqueous colloidal solutions of nanoparticles with different concentrations has been studied.

**Methods:** The methods of studying the characteristics of plasma and acoustic oscillations induced by optical breakdown are applied. The methods of assessing the concentration of hydrogen peroxide and hydroxyl radicals, the amount of long-lived reactive species of protein and 8-oxoguanine are applied.

**Results:** It has been established that in the process of laser fragmentation of selenium nanoparticles at a wavelength of 532 nm, corresponding to the maximum absorption of selenium, the highest probability of breakdown, the number of plasma flashes, their luminosity and the amplitude of acoustic signals are achieved at concentrations of the order of 109 NPs/mL. It has been shown that the use of selenium nanoparticles of various shapes and structures leads to a change in the photoacoustic signal during laser-induced breakdown. When crystalline selenium nanoparticles are irradiated, the intensity of the photoacoustic response during breakdown turns out to be greater (1.5 times for flash luminosity and 3 times for acoustics) than when amorphous particles are irradiated at the same concentration. It has been shown that selenium nanoparticles exhibit significant antioxidant properties. Selenium nanoparticles effectively prevent the formation of reactive oxygen species (ROS) during water radiolysis, eliminate radiation-induced long-lived reactive species of protein, and reduce the radiation-chemical yield of a key marker of oxidative DNA damage - 8-oxoguanine.

**Discussion:** In general, the intensity of processes occurring during laser fragmentation of amorphous and crystalline selenium nanoparticles differs significantly. The antioxidant properties are more pronounced in amorphous selenium nanoparticles compared to crystalline selenium nanoparticles.

## 1 Introduction

Due to the rapid development and widespread use of radioisotopes and radiation technologies in various fields of human activity, for example, in nuclear energy, medicine, industry and agriculture, the likelihood of exposure to ionizing radiation on the human body and the environment increases (Cheng et al., 2023; Duarte et al., 2023; Paulillo et al., 2023). Ionizing radiation causes radiolysis of water molecules, which leads to excessive production of ROS (Sharapov et al., 2021) and causes damage to critical cellular components such as DNA, RNA, proteins and lipids (Gudkov et al., 2009). Irreversible damage to biomolecules is often the cause of cell death due to necrosis and apoptosis (Dehghan et al., 2023). In this regard, the development of radioprotective agents to reduce the harmful effects of ionizing radiation, especially in the conditions of nuclear accidents and radiation therapy, was and is of great importance (Sharapov et al., 2019). Research into the mechanisms of radiation injury and protective measures is crucial for the development of scientific knowledge in the field of radioprotection (Wojcik and Harms-Ringdahl, 2019). To date, a number of radioprotective drugs have been developed, including sulfhydryl radioprotectors, nitroxides, natural antioxidants, protein drugs, selenium-containing compounds, including nanosized selenium (Liu et al., 2023).

It should be noted that nanosized selenium has a wide range of biomedical applications (Karthik et al., 2024). Its effect on reducing oxidative stress levels is well known (Varlamova et al., 2021a). In this regard, selenium and selenium-containing nanoparticles are increasingly being studied as radioprotective agents (Karami et al., 2017; Gudkov et al., 2023; Azmoonfar et al., 2024). The size of selenium nanoparticles plays an important role in their biological activity (Varlamova et al., 2021b). It has been shown that the ability of Se particles ranging in size from 5 to 200 nm to directly absorb free radicals *in vitro* depends on the particle size (Peng et al., 2007). However, in most studies, mainly amorphous selenium is studied (Zhang et al., 2001; Guleria et al., 2021; Li et al., 2024). This is mainly due to the specific methods used to synthesize the particles, and there is insufficient information in the literature about the biological effects of nanosized selenium in other forms (Zhang et al., 2023).

It is known that selenium has about 11 modifications, of which 7 are crystalline and 4 are non-crystalline, which include two amorphous forms, liquid and glassy selenium (Minaev et al., 2005). Among the crystalline modifications of selenium, trigonal selenium (t-Se) is distinguished, which has a number of unique optical and electrical properties that are associated with its band structure (Day, 1971). Selenium in trigonal form forms nanocrystalline rods with a thickness of several tens to hundreds of nanometers and a length of up to several microns (Qi and Cheng, 2019). It is assumed that the shape of trigonal selenium nanoparticles will also have a noticeable effect on their biological properties (Abbasi et al., 2023). Therefore, the purpose of this work will be to assess the influence of the structure and shape of selenium particles on the antioxidant and protective properties of selenium particles.

## 2 Materials and methods

### 2.1 Preparation and characterization of selenium nanoparticles

Selenium nanoparticles were obtained using laser ablation and laser fragmentation techniques. A polished target made of polycrystalline pure Se (99.99%) was placed in a 30 mL glass cell and placed at the bottom of the cell. The thickness of the liquid layer between the surface of the target and the liquid was 2–3 mm. Deionized water and chemically pure propanol-2 were used as working fluids. An Nd:YAG laser NL300 (Ekspla, Vilnius, Lithuania) with the following parameters was used as a source of laser radiation: pulse duration τ = 3.6 ns, frequency υ = 1 kHz, wavelength λ = 532 nm, pulse energy ε = 2 mJ (Turovsky et al., 2022). During irradiation, the beam was moved along the target surface using a galvanomechanical scanner LScanH (Ateko-TM, Moscow, Russia) and an F-Theta lens with a focal length of 90 mm. The spot size in the waist was 100 µm. The energy density of laser radiation on the target surface was 25 J/cm^2^. The beam trajectory consisted of several parallel lines inscribed in a 1 × 1 cm^2^ square. The radiation speed was 3,000 mm/s. Laser ablation of the target occurred within 30 min.

In laser fragmentation of colloidal solutions of selenium nanoparticles, a colloidal solution obtained as a result of laser ablation was used. The colloid was placed in a glass cuvette with a transparent bottom, the radiation was supplied using a reflecting mirror into the cuvette from below and focused at a distance of 1–2 cm from the bottom to avoid radiation scattering on the bubbles of the resulting gas. Laser ablation of colloids occurred within 30–45 min (Gudkov et al., 2020).

Se nanoparticles obtained by laser ablation and fragmentation in isopropanol were transferred to deionized water by centrifugation and washing in various chemical solvents. Nanoparticles were initially sedimented using an LMC-4200 centrifuge (Biosan, Riga, Latvia). Centrifugation was carried out at 4,200 rpm (3,160 g) for 15 min. Propanol-2 was taken from the solution with precipitated nanoparticles and a chemically pure solution of carbon tetrachloride (CCl_4_) was added. The resulting solution was placed in an ultrasonic bath (ultrasound power 20 W) for 10 min. Similar procedures of centrifugation, solution replacement and ultrasound were carried out for dimethyl sulfoxide (C_2_H_6_OS), chloroform (CHCl_₃_), acetone (C_3_H_6_O). At the last stage, the solvent was replaced with deionized water.

A Libra 200 FE HR transmission electron microscope (TEM) (Carl Zeiss, Jena, Germany) was used to obtain TEM-images of particles and study their morphology. Gold microscopic grids were used to prepare SeNPs for TEM microscopy. A DC24000 analytical centrifuge (CPS Instruments, Oosterhout, Netherlands) and a Zetasizer Ultra (Malvern Panalytical, Malvern, United Kingdom) were used to study the nanoparticle size distribution and determine the nanoparticle concentration. The absorption spectrum of colloidal solutions of selenium nanoparticles was studied using a two-channel spectrometer UV-3600 Series (Shimadzu, Japan) (300–1,600 nm). The spectra were measured in quartz cuvettes with a volume of 3 mL. The absorption spectra of deionized water were used as reference spectra. The structure of selenium nanoparticles was studied using X-ray diffraction patterns obtained using a Bruker AXS P4 diffractometer (Bruker, Billerica, MA, United.States).

### 2.2 Experimental setup used to register photoacoustic signals

In experiments to study photoacoustic signals observed during optical breakdown, a similar experimental scheme was used, which was used in our earlier works, for example, in (Baimler et al., 2021). To irradiate colloidal solutions of nanoparticles, laser with the following parameters was used (λ = 532 nm, τ = 3.6 ns, ν = 1 kHz, ε = 2 mJ, beam diameter 50 μm). The colloidal solution of selenium nanoparticles was irradiated for 2 min. During the irradiation time, photographs of breakdown plasma flashes were taken and the spectra of acoustic signals were recorded.

The plasma formed during optical breakdown was photographed using a Canon EOS 450D digital camera (macro mode, grayscale, shutter speed 100 m). The number of flashes in the image and the total luminosity of the flash were calculated when processing photographs using the automatic program LaserImage; a detailed description and operating principle of the program are given in (Nagaev et al., 2022). The value of the integral plasma luminosity was also recorded using a DET10A2 silicon photodetector (Thorlabs, Newton, New Jersey, United.States).

The acoustic spectrum of the laser breakdown was recorded using a film piezoelectric sensor integrated into the cell. The sensor plane was located parallel to the scanning line of laser radiation. The acoustic sensor is connected to a digital oscilloscope (GW Instek GDS-72204E, GW Instek, Xinbei, Taiwan). The oscilloscope was synchronized with the laser using a pin diode so that the laser signal triggered the sweep of the digital oscilloscope. A specially developed LaserCav program was used to analyze the data. Measurements of acoustic signals and acoustic processes occurring in our system have been described in detail previously (Baimler et al., 2020a).

### 2.3 X-ray irradiation of solutions

The solutions were irradiated at room temperature on a therapeutic X-ray machine RUT-15 (Mosrentgen, Russia) with a dose rate of 0.1 Gy/min (7 mA, 200 kV, focal length 105 cm), 4.5 Gy/min (17 mA, 200 kV, focal length 19.5 cm) and 1.0 Gy/min (15 mA, 200 kV, focal length 37.5 cm).

### 2.4 Measurement of H_2_O_2_ and OH-radicals, 8-oxoguanine and long-lived reactive species of protein

The H_2_O_2_ concentration was determined by enhanced chemiluminescence in the luminol/4-iodophenol/peroxidase system. The intensity of chemiluminescence was measured using a Beta-1 liquid scintillation counter (Medapparatura, Russia) in photon counting mode; calibration was carried out using H_2_O_2_ samples of known concentration. Details of the procedure were published in (Shcherbakov et al., 2020). The concentration of OH radicals was determined using coumarin-3-carboxylic acid. Fluorescence intensity was measured on a Jasco 8,300 spectrofluorimeter (Jasco, Japan) with λ_ex_ = 400 nm and λ_em_ = 450 nm. Before measurement, the pH was adjusted to 8.5 using Tris-HCl buffer. Details of the procedure were published in (Sergeichev et al., 2021). Measurement of 8-oxoguanine (8-oxoG) in DNA was performed by enzyme-linked immunosorbent assay. Details of the procedure were published in (Chernikov et al., 2007). Long-lived reactive species of protein were studied by measuring the X-ray induced chemiluminescence of protein solutions using a specially designed highly sensitive Biotoks-7AM chemiluminometer (Ekon, Russia). Measurements were carried out in the dark at room temperature in 20 mL plastic polypropylene vials for liquid scintillation counting. Details of the procedure were published in (Sevostyanov et al., 2020).

### 2.5 Statistics analysis

Mean and standard error of the mean (SEM) were calculated for 10 measurements for most variables. Correlation coefficient was calculated, and the means from the different groups were compared by Student’s unpaired *t*-test. Statistical significance was assigned to *P* < 0.05. Statistically significant differences between the irradiation control group and the other groups are marked by asterisks.

## 3 Results

### 3.1 Morphology of obtained selenium nanoparticles

**FIGURE 1:**
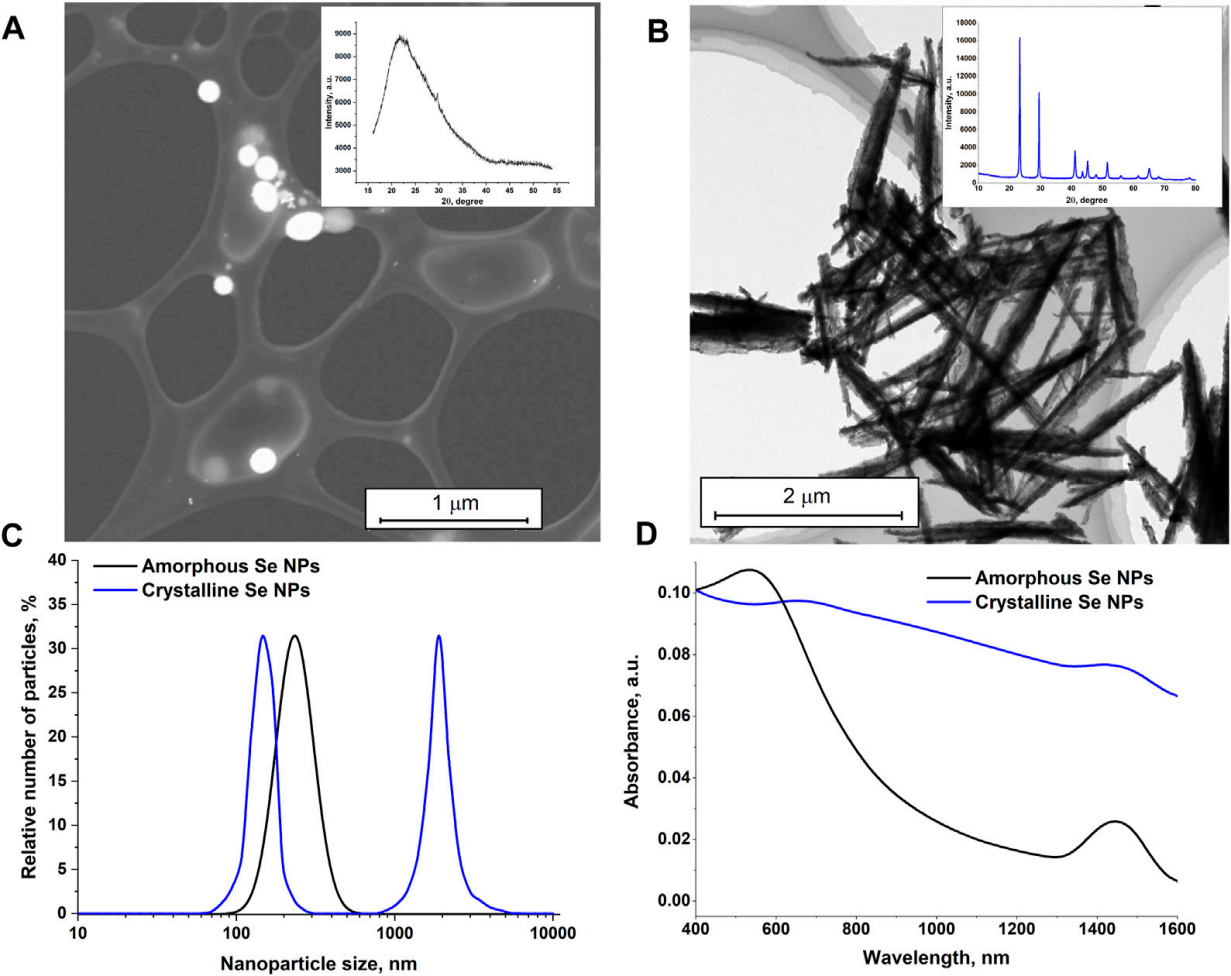
Characteristics of selenium nanoparticles obtained as a result of laser ablation in deionized water and isopropanol. TEM images of selenium nanoparticles obtained using the laser ablation technique of a solid selenium target in deionized water **(A)**, in isopropanol **(B)**. Insert: X-ray diffraction patterns of selenium particles corresponding to TEM photographs. **(C)** – Size distribution for amorphous (obtained in deionized water) and crystalline (obtained in isopropanol) selenium particles. **(D)** – Absorption spectrum of colloids of nanoparticles of amorphous and crystalline selenium particles.

Figure 1 shows TEM images of selenium nanoparticles obtained as a result of laser ablation in deionized water (Figure 1A) and isopropanol (Figure 1B). It was found that the nanoparticles obtained in deionized water have a spherical shape, with the size of the nanoparticles being hundreds of nanometers (Figure 1A). Particle X-ray diffraction data (Inset in Figure 1A) indicate the amorphous structure of selenium (α-Se) nanoparticles. Nanoparticle size data obtained from TEM are consistent with size distributions obtained from the CPS Analytical Centrifuge and DLS Particle Analyzer (Figure 1C). The largest number of Se nanoparticles in the distribution is around 200 nm in size (Figure 1C).

From the TEM images, it appears that the selenium nanoparticles prepared in isopranolol are nanorod-shaped (Figure 1B) and have a crystal structure consistent with trigonal selenium (t-Se) (Inset in Figure 1B). The size distribution of nanoparticles is bimodal (Figure 1C). One of the maxima in the size distribution corresponds to 150 nm, the other to 1800 nm.

The absorption spectra of colloidal solutions of amorphous and crystalline selenium nanoparticles obtained using laser ablation and laser fragmentation techniques in various liquids were studied. It was shown that the main distinctive feature of the absorption spectrum of elongated selenium nanoparticles is an increase in absorption in the red region of the spectrum. For spherical nanoparticles, the maximum absorption occurs at approximately 550 nm, as shown in Figure 1D. In the infrared range, the absorption of radiation by spherical nanoparticles is much less.

### 3.2 Influence of the morphology of selenium nanoparticles on laser-induced breakdown plasma and shockwaves

**FIGURE 2:**
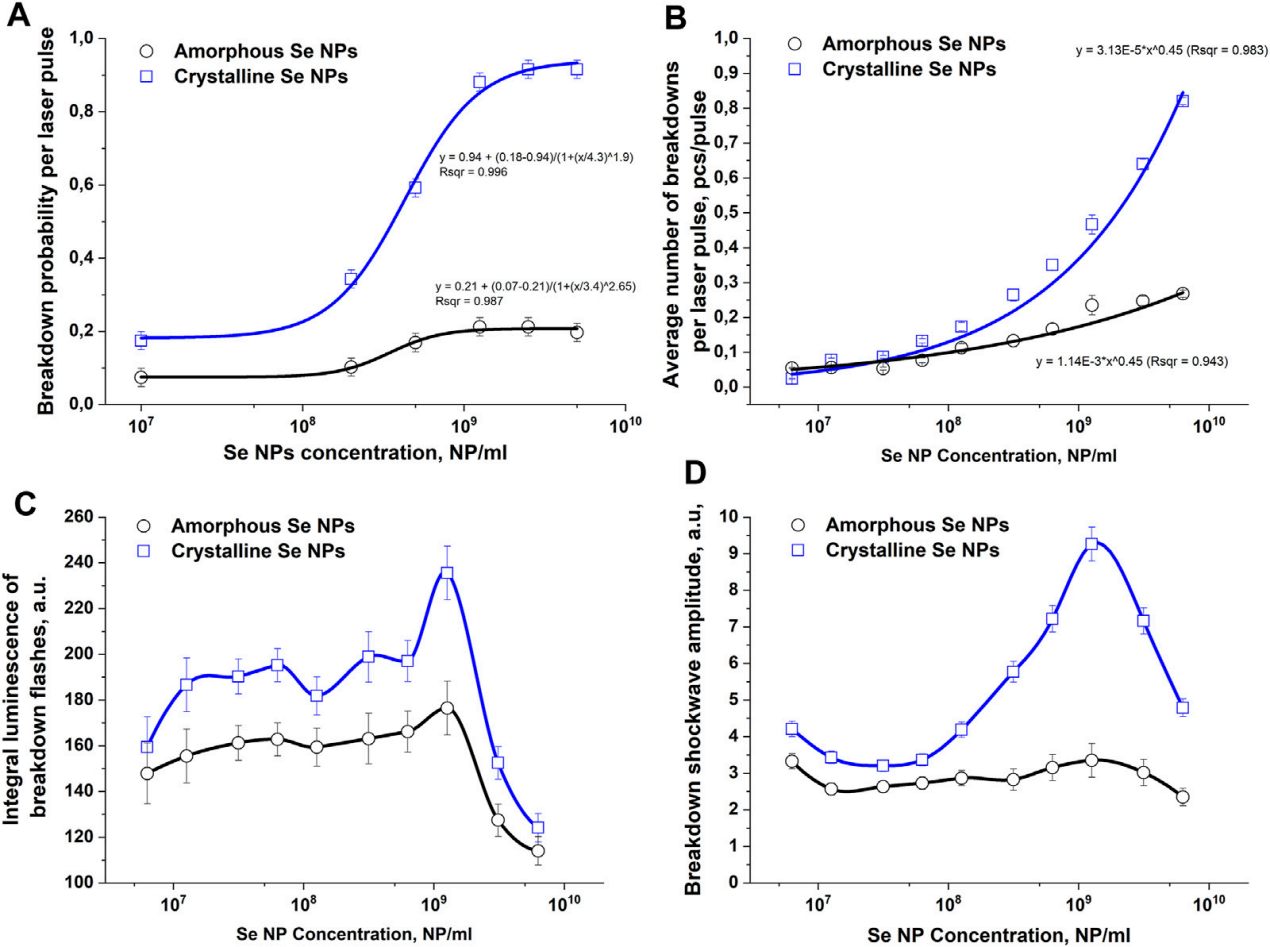
The influence of amorphous and crystalline selenium nanoparticles on the intensity of optical and acoustic effects observed during optical breakdown of aqueous colloids of these nanoparticles. **(A)** – Effect of the concentration of selenium nanoparticles of different structures in solution on the probability of optical breakdown during laser irradiation of a colloid. **(B)** – Dependence of the average number of breakdowns per laser pulse on the concentration of amorphous and crystalline selenium nanoparticles. **(C)** – Dependence of the plasma flash luminosity on the concentration of amorphous and crystalline selenium nanoparticles in solution. **(D)** – Change in the amplitude of the acoustic signal depending on the concentration and structure of selenium particles; The values presented in the graphs are averaged over 10 measurements. Error bars denote the standard error of mean. A spline was used to connect the points in Figures C and **(D)**

The influence of the structure of selenium nanoparticles on the probability of optical breakdown, the number of breakdowns per laser pulse, the value of the integral plasma luminosity and the amplitude of acoustic signals during irradiation and breakdown of colloids of selenium nanoparticles in aqueous solutions was studied, Figure 2. It has been shown that when colloidal solutions of spherical particles of amorphous and crystalline selenium are irradiated, the probability of optical breakdown increases monotonically with increasing concentration of nanoparticles (Figure 2A). When irradiating amorphous particles, the maximum breakdown probability per laser pulse is approximately 0.20–0.22 and is achieved at nanoparticle concentrations of 10^9^ NPs/mL. When a colloid with selenium nanorods is irradiated, the probability of breakdown with a change in the concentration of nanoparticles changes approximately 4 times from 0.2 at concentrations of 10^7^–10^8^ NPs/mL to 0.9 for concentrations 10^9^–10^11^ NPs/mL.

The change in the number of individual breakdowns per laser pulse depending on the concentration of Se nanoparticles in the colloidal solution is presented in Figure 2B. In the concentration range of Se nanoparticles from 10^7^ to 10^8^ NPs/mL, the average number of breakdowns is approximately 0.1 pcs/pulse for both amorphous and crystalline particles. As the concentration of amorphous selenium particles in the colloid increases to 10^9^ NPs/mL, the number of breakdowns per laser pulse monotonically increases and reaches a maximum value of approximately 0.25 pieces/pulse. When colloids of crystalline selenium nanoparticles are irradiated at a concentration of 10^9^ NPs/mL, the average number of breakdowns per laser pulse is approximately 0.35 pcs/pulse. With a further increase in the concentration of crystalline Se nanoparticles, the average number of breakdowns per laser pulse reaches approximately 0.85 pcs/pulse at concentration values of 10^10^ NPs/mL, which is approximately 3.5 times greater than for amorphous particles.

The integral plasma luminosity in the studied range of nanoparticle concentrations changes nonmonotonically (Figure 2C). With an increase in the concentration of nanoparticles from 10^7^ NPs/mL to 10^9^ NPs/mL, the integral brightness gradually increases from values of 150–160 rel. units to a maximum at a nanoparticle concentration of 10^9^ NPs/mL. The value of the integral glow intensity of the breakdown plasma at this concentration for crystalline particles (240 rel. units) turns out to be approximately 40% greater than for amorphous particles (170 rel. units). With a subsequent increase in the concentration of nanoparticles, the integral luminosity of the breakdown plasma flash begins to monotonically decrease to values of 120 rel. units at a concentration of 10^10^ NPs/mL for amorphous and crystalline particles.

Changes in the intensity of the acoustic response of the breakdown plasma depending on the concentration and structure of selenium nanoparticles in the colloidal solution are presented in Figure 2D. It has been shown that the amplitude of ultrasonic vibrations increases, reaches a maximum and then decreases with a gradual increase in the concentration of selenium particles in the irradiated colloid. In the case of breakdown of amorphous and crystalline selenium particles in a colloid in the concentration range from 10^7^ NP/mL to 10^8^ NP/mL, the amplitude of the acoustic signals does not change significantly. After a further increase in the concentration of nanoparticles to 10^9^ NPs/mL, the most intense acoustic signals are observed in the irradiated solution of crystalline particles, but the amplitude of acoustic vibrations does not change significantly when amorphous particles are irradiated. Starting from a concentration of 10^9^ NPs/mL to 10^10^ NPs/mL, the intensity of shock waves during breakdown begins to decrease for both amorphous and crystalline selenium particles.

### 3.3 Influence of the structure of selenium nanoparticles on radiation-induced generation of reactive oxygen species and damage of biomacromolecules

**FIGURE 3:**
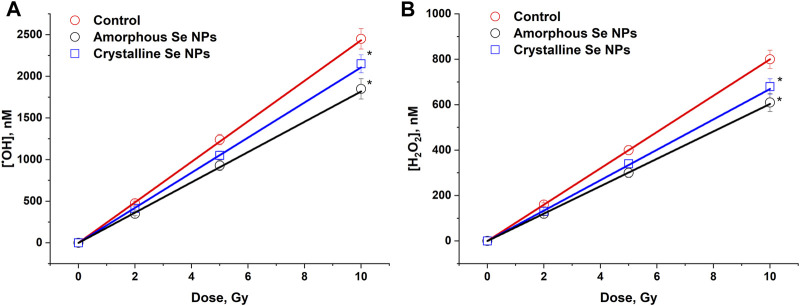
The influence of amorphous and crystalline selenium nanoparticles (100 mg/L) on the formation of OH-radicals **(A)** and H_2_O_2_ **(B)** in water under the influence of X-ray irradiation in doses of 2–10 Gy. The data for 10 measurements were used. The values are significantly different from control values at *P* < 0.05 (*).

The effect of Se nanoparticles with amorphous and crystalline structure on the generation of OH-radicals in the X-ray irradiated colloid is shown in Figure 3A. The amount of OH- radicals was linearly dependent on the dose both in the control (in the absence) and in the presence of selenium nanoparticles. A decrease in the formation of OH-radicals by 10%–15% compared to the control sample was observed in the presence of selenium nanoparticles of amorphous and crystalline structure. The radiation chemical yield (G) of OH-radicals in the absence of Se nanoparticles was 2.42 ± 0.12 molecules/100 eV (0.24 μM/Gy). In the presence of amorphous Se nanoparticles, the value G = 1.85 ± 0.09 molecules/100 eV (0.18 μM/Gy), in the presence of crystalline Se nanoparticles G = 2.15 ± 0.11 molecules/100 eV (0.21 μM/Gy). These patterns were also observed at low doses of 0.1 Gy.

The effect of amorphous and crystalline selenium NPs on the formation of H_2_O_2_ in water under X-ray irradiation was studied (Figure 3B). It was shown that the amount of H_2_O_2_ was linearly dependent on the dose both in the control (in the absence) and in the presence of selenium nanoparticles. A 15%–25% reduction in the formation of hydrogen peroxide was observed upon irradiation of colloids of amorphous and crystalline selenium NPs. The radiation-chemical yield for H_2_O_2_ was 0.80 ± 0.04 molecules/100 eV (80 nM/Gy) in the absence of selenium nanoparticles, while when amorphous particles were irradiated, the G value was 0.61 ± 0.03 molecules/100 eV (61 nM/Gy) and G = 0.68 ± 0.03 molecules/100 eV (68 nM/Gy) in the presence of crystalline particles. These values were maintained over a small dose range (0.1 Gy).

**FIGURE 4:**
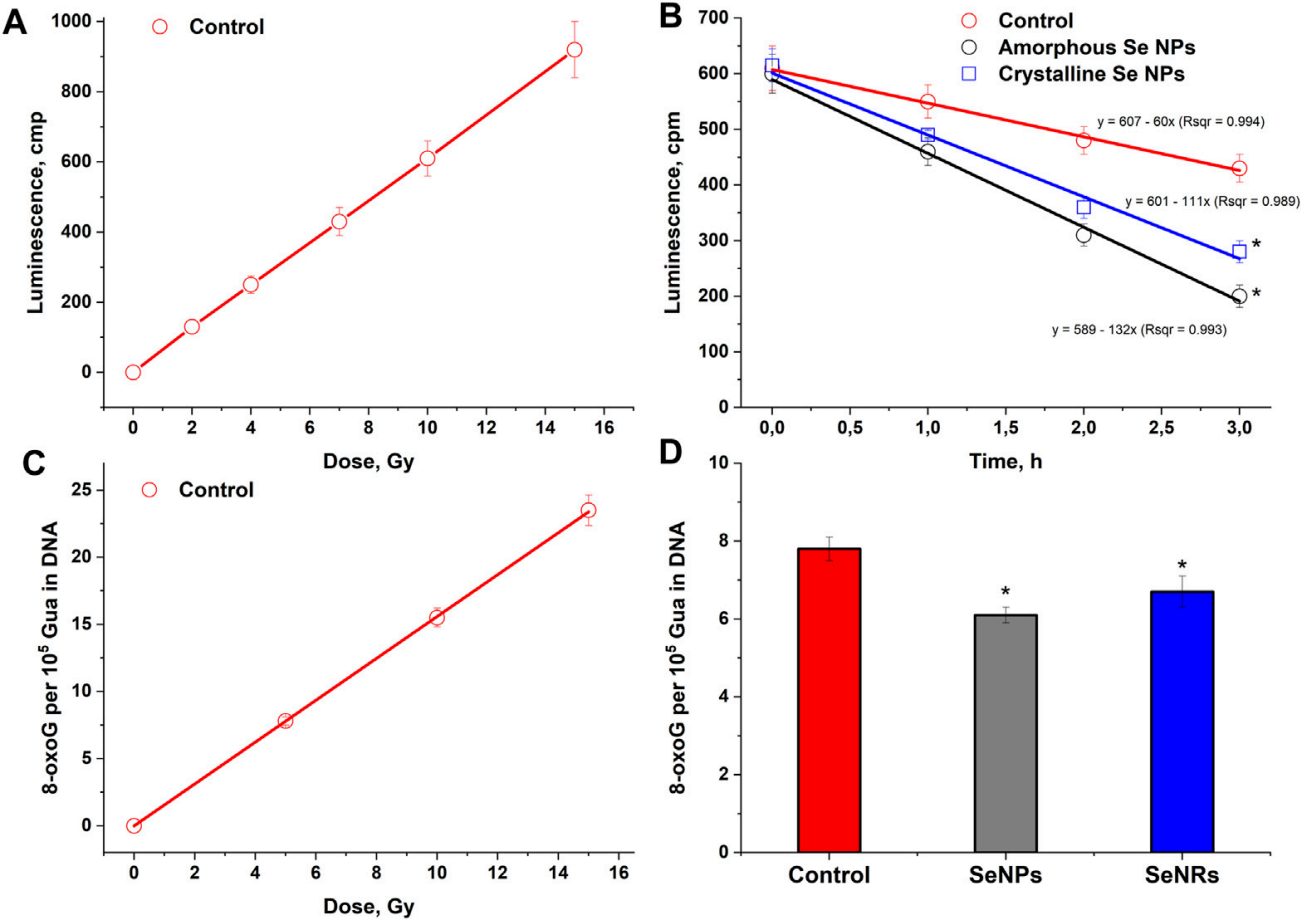
Effect of selenium nanoparticles with amorphous and crystalline structure on radiation-induced damage to proteins and DNA. **(A)** – Effect of the absorbed dose of ionizing radiation on the luminescence intensity of an aqueous solution of bovine serum albumin (1 g/L). Measurements were taken 30 min after exposure to X-rays. **(B)** – Change in the luminescence intensity of aqueous solutions of bovine serum albumin (1 g/L) containing or not containing amorphous or crystalline selenium nanoparticles (100 mg/L) after exposure to X-ray radiation at a dose of 7 Gy. Background luminescence values were subtracted from the results. **(C)** – Effect of the absorbed dose of ionizing radiation on the amount of 8-oxoG in DNA. **(D)** – Effect of selenium nanoparticles of various structures on the formation of 8-oxoG in DNA *in vitro* upon irradiation at a dose of 7 Gy. The data for 10 measurements were used. The values are significantly different from control values at *P* < 0.05 (*).

The effect of the absorbed dose of ionizing radiation on the luminescence intensity of an aqueous solution of bovine serum albumin is presented in Figure 4A. It has been shown that the intensity of luminescence induced by long-lived reactive species of protein linearly depends on the absorbed dose of X-ray radiation in the range of 2–15 Gy. The change in luminescence intensity dependence on time in aqueous solutions of bovine serum albumin (1 g/L) containing or not containing amorphous or crystalline selenium nanoparticles (100 mg/L) after exposure to X-ray radiation at a dose of 7 Gy is presented in Figure 4B. The average half-life of long-lived reactive species of protein is about 5 h. The addition of crystalline selenium nanoparticles reduces the half-life of long-lived reactive species of proteins to 3 h. The addition of amorphous selenium nanoparticles reduces the half-life of long-lived reactive species of proteins to 2.5 h. Thus, the elimination of long-lived reactive species of proteins in the presence of selenium nanoparticles occurs 1.6–2.0 times faster.

The dose dependence of selenium nanoparticles of amorphous and crystalline structures, affecting the generation of 8-oxoG in a DNA solution under the influence of X-ray radiation, was studied (Figure 4C). The amount of 8-oxoG in the presence and absence of selenium nanoparticles depends linearly on the X-ray dose. A 20% reduction in 8-oxoG formation in DNA was observed in the presence of amorphous selenium nanoparticles (Figure 4D). In the presence of crystalline particles, the amount of 8-oxoG in DNA is reduced by approximately 15%. The radiation chemical yield values for 8-oxoG in the control sample, the sample with amorphous particles and the sample with crystalline particles were 0.78; 0.66 and 0.62 molecules/100 eV, respectively.

## 4 Discussion

In this work, preparations of selenium nanoparticles with an amorphous (α-Se) and crystalline (*t*-Se) structure were obtained (Figure 1). It has been established that nanoparticles with an amorphous crystalline structure have different morphologies and spectral properties. It has been established that the probability of optical breakdown; number of breakdowns induced by one pulse; the intensity of plasma glow and acoustic vibrations depend on the concentration, shape and structure of selenium nanoparticles in the irradiated solution (Figure 2). An increase in the concentration of amorphous and crystalline selenium nanoparticles in the irradiated aqueous solution leads to an increase in the probability of optical breakdown, which is typical for the irradiation of most nanoparticles. However, the probability of breakdown in colloids of crystalline selenium nanorods in the studied concentration range is higher compared to amorphous selenium particles. Other experimentally observed effects—an increase in the number of breakdowns per pulse, an increase in the glow intensity of the plasma flash, and an increase in the intensity of the acoustic signal—are likely the result of a change in the breakdown probability.

Crystalline selenium of the trigonal modification is a semiconductor with a band gap E_g_ = 1.89 eV, and the ionization energy of trigonal selenium is approximately equal to the band gap, since selenium is characterized by the effect of impact ionization and avalanche-like electron multiplication (Darbandi and Rubel, 2013). For amorphous selenium, the band gap is estimated to be E_g_ = 2–2.3 eV (Reznik et al., 2007). The difference in ionization energy for amorphous and trigonal selenium, together with the presence of electrons in the conduction band of trigonal selenium, which contribute to the development of an electron avalanche, can explain the difference in the probability of optical breakdown (Figure 2A) and the number of optical breakdowns induced by one laser pulse (Figure 2B).

In works (Baimler et al., 2020a; Baimler et al., 2021; Simakin et al., 2023) devoted to the study of the influence of different concentrations of nanoparticles on the physical and chemical processes occurring during laser breakdown of colloids, it was noted that the probability of breakdown, plasma glow intensity, amplitude of acoustic signals, and the rate of formation of chemical dissociation products are greatest at concentration about 10^9^–10^10^ NPs/mL. It has been shown that the value of the optimal concentration does not depend on the type of nanoparticle material (Baimler et al., 2020b). However, it is worth noting that the optical breakdown of nanoparticle colloids in all these works was initiated using nanosecond laser radiation at a wavelength of 1,064 nm. Recently, using a high-speed streak camera, it was shown that the parameters of individual plasma flares weakly depend on the radiation energy density (Baimler et al., 2024). It was suggested that the breakdown plasma quite quickly (during the duration of the laser pulse) transitions to a critical regime, in which the absorption of laser radiation by the plasma stops and the plasma screens the radiation with a frequency equal to the plasma frequency:

$$\omega_{P}=\sqrt{\frac{4\pi n_{e}e^{2}}{m}}$$

where, the concentration of plasma electrons n_e_ is determined, at the initial moments of breakdown development, mainly by electrons formed as a result of ionization of atoms of the nanoparticle material (for a selenium particle with a size of 100 nm, the number of electrons formed as a result of ionization is n_e_ ∼ 10^9^ pcs.) and further expansion of the plasma reaches critical values (n_e_^cr^ ≈ 1 × 10^21^ cm^−3^), due to the ionization of atoms of the liquid surrounding the nanoparticle. For wavelengths of 1,064 nm and 532 nm, the critical concentration of electrons in the plasma is n_e_^cr^ ≈ 1 × 10^21^ cm^−3^ and n_e_^cr^ ≈ 4 × 10^21^ cm^−3^, respectively, i.e., for shorter wavelengths, the value of the critical concentration is greater, and the plasma in the critical regime is more dense, which is observed at lower particle concentrations. It follows that the breakdown plasma is more localized and has a lower temperature during irradiation and breakdown of the colloid at a wavelength of 532 nm. Perhaps the proposed pattern explains the difference in the observed values of “effective” concentrations of nanoparticles at which the greatest photoacoustic response of laser-induced plasma is observed when irradiated at different wavelengths (Figure 2).

Radiolysis is usually understood as the process of dissociation of molecules caused by the action of ionizing radiation, and the formation of new chemical compounds, both radical and neutral, is observed. During the radiolysis of water, for example, the formation of ROS is observed (Ward, 1975). It should be noted that ROS are also formed under the action of other physicochemical factors, but the mechanism of their formation is significantly different (Lyakhov et al., 2022; Shcherbakov, 2022; Stepanov and Shcherbakov, 2023). ROS are typically strong oxidizing agents or highly reactive free radicals. For 100 electron-volts of absorbed energy of ionizing radiation in an aquatic environment, on average: 2.4 OH-radicals are formed; 2.8 solvated electrons; 0.4 hydrogen atoms; 0.8 molecules of H_2_O_2_; 0.4 molecules of H_2_ and significantly less than other compounds (Ward, 1988), which is largely consistent with the results obtained (Figure 3). An increase in the intracellular concentration of ROS above the level of antioxidant protection causes “oxidative stress” (Chaudhary et al., 2023), which is accompanied by life-threatening processes of cells, such as lipid peroxidation (Geng et al., 2023), oxidative modification of proteins (Khramova et al., 2024) and nucleic acids (Marmiy and Esipov, 2015).

Proteins are an essential part of food and the basis of life (Anupama and Ravindra, 2000). Proteins make up 10%–20% of the wet weight and 50%–80% of the dry weight of the cell (Day et al., 2022). In the middle of the last century, it was shown that when exposed to ionizing radiation, along with short-lived ROS that arise during the radiolysis of water, long-lived reactive species of proteins are also formed (Kayushin et al., 1960). Their radical nature was later established. It has been shown that when aqueous solutions of albumin are exposed to gamma rays, protein radicals are formed with half-lives at room temperature of more than 20 h (Yoshimura et al., 1993). It has been shown that such long-lived reactive species of proteins can be secondary sources of ROS formation (Ivanov et al., 2017) and can damage DNA and other biomolecules (Bruskov et al., 2012). It has been shown that a number of antioxidants are able to effectively eliminate long-lived reactive species of proteins. In particular, such properties are manifested by inosine, guanosine (Gudkov et al., 2007), vitamin C (Koyama et al., 1998), epigallocatechin gallate (Kumagai et al., 2002), gallic (Koyama et al., 1998) and uric (Pietraforte and Minetti, 1997) acids. This work shows for the first time that selenium nanoparticles can also effectively eliminate long-lived reactive species of proteins (Figures 4A, B). Moreover, the effectiveness exhibited by selenium nanoparticles is comparable to the effectiveness of low molecular weight antioxidants.

Damage to DNA molecules is often one of the main causes of post-radiation death of living systems (Ponomarev et al., 2023). A significant part (about 70%–80%) of DNA damage caused by radiation is formed due to ROS formed during the radiolysis of water, and only 20%–30% due to the direct absorption of high-energy quanta of ionizing radiation by target molecules (Gudkov et al., 2015). The most common oxidative DNA damage is 8-oxoguanine (Li and Wang, 2024). This oxidative modification of guanine has ambiguous coding properties and can lead to the appearance of a point mutation in DNA (Yudkina et al., 2024). For every 100 electron volts of absorbed ionizing radiation energy, an average of 0.78 molecules of 8-oxoguanine are formed in polyguanine (Ward, 1988), which is substantially consistent with the results obtained (Figures 4C, D).

Thus, it has been shown that selenium nanoparticles exhibit significant antioxidant properties. Selenium nanoparticles effectively prevent the formation of ROS during water radiolysis, eliminate radiation-induced active forms of proteins, and reduce the radiation-chemical yield of a key marker of oxidative DNA damage - 8-oxoguanine. It can be assumed that the selenium nanoparticles we obtained will have significant radioprotective properties.

## Data Availability

The raw data supporting the conclusions of this article will be made available by the authors, without undue reservation.
